# Effects of curcumin supplementation on vitamin D levels in women with premenstrual syndrome and dysmenorrhea: a randomized controlled study

**DOI:** 10.1186/s12906-022-03515-2

**Published:** 2022-01-22

**Authors:** Leyla Arabnezhad, Mahtab Mohammadifard, Ladan Rahmani, Zahra Majidi, Gordon A. Ferns, Afsane Bahrami

**Affiliations:** 1 Zahedan University of Medical Sciences, Zahedan, Iran; 2 Infectious Diseases Research Center, Birjand University of Medical Sciences, Birjand, Iran; 3 Student Research Committee, Birjand University of Medical Sciences, Birjand, Iran; 4 Brighton & Sussex Medical School, Department of Medical Education, Falmer, Brighton, Sussex, BN1 9PH UK; 5 Clinical Research Development Unit of Akbar Hospital, Faculty of Medicine, Mashhad University of Medical Sciences, Mashhad, Iran; 6 Clinical Research Development Unit, Imam Reza Hospital, Faculty of Medicine, Mashhad University of Medical Sciences, Mashhad, Iran

**Keywords:** Menstruation, Triglycerides, Turmeric, Aspartate aminotransferase, Bilirubin

## Abstract

**Background:**

Vitamin D has an established role in female reproduction. There is also evidence for an association between vitamin D levels and menstrual problems such as premenstrual syndrome (PMS) and dysmenorrhea. Curcumin, is a bioactive polyphenol constituent of turmeric, that can potentially interact with vitamin D receptors and its molecular targets. This study evaluated the effects of curcumin on vitamin D levels in young women with PMS and dysmenorrhea.

**Methods:**

In this randomized, triple-blind, placebo-controlled trial, women with PMS and dysmenorrhea were divided randomly into experimental and control groups to receive one capsule (500 mg of curcuminoid+ 5 mg piperine, or placebo) daily, from approximately 7 days before until 3 days after menstruation for three consecutive menstrual cycles. Serum vitamin D levels, renal function, and liver enzymes were also measured before and after intervention.

**Results:**

A total of 76 subjects (38 in each group) were recruited into the trial. Curcumin significantly increased the median (IQR) serum levels of vitamin D [from 12.8 ng/ml (7.0–24.6) to 16.2 ng/ml (6.4–28.8); *P* = 0.045], compared with placebo [from 18.6 ng/ml (2.2–26.8) to 21.3 ng/ml (5.2–27.1); *P* = 0.17]. Serum levels of aspartate aminotransferase and direct bilirubin were reduced by the end of trial in the curcumin group (*p* < 0.05), but did not change significantly in the control group (*p* > 0.05). Finally, no significant differences in levels of fasting blood glucose were detected between curcumin and placebo groups.

**Conclusion:**

Curcumin supplementation in women with PMS and dysmenorrhea led to a significant improvement of vitamin D, liver function enzyme test, but did not affect blood glucose.

**Trial registration:**

The trial was registered on Iranian Registry of Clinical Trials registry (Trial ID: IRCT20191112045424N1 on 23 January 2020; available at https://www.irct.ir).

## Introduction

Premenstrual syndrome (PMS) and dysmenorrhea are common cyclical and recurrent gynecologic complications of women in the reproductive age, and can adversely affect their wellbeing and quality of life. PMS is defined by a complex combination of somatic and psychological bothering symptoms that happens within the luteal phase which lasting from ovulation to the beginning of the menstrual hemorrhage [1].

Dysmenorrhea characterized by spasmodic cramping pain in the lower abdomen, in the absence of pelvic problems, often happens just pre or at the onset of menstrual bleeding and persists for 8–72 h. The pathogenic mechanism behind dysmenorrhea are not been fully understood, but may be because of the increase in the generation of prostaglandins and leukotrienes [2].

No definitive treatment for PMS and dysmenorrhea has been established so far; therapies has been symptomatic, administrating such medications as non-steroid anti-inflammatory drugs (NSAIDs), antidepressants, combined oral contraceptives, and herbal medicine [3–5]. Complementary and alternative medical therapies are sometimes preferred by women for alleviating of menstrual associated pains. Although, the effectiveness and safety of this type of treatment has not be comprehensively investigated in randomized controlled trials.

The potential role of vitamins and mineral status in the etiology of common features of PMS and dysmenorrhea, and possibly a putative mechanism for the preventing and/or treating of systemic menstrual problems has been reported [1, 2, 6]. There are several reports indicating an inverse relationship between vitamin D (Vit D) status and risk of depressive symptoms [7, 8], fibromyalgia [9], and uterine leiomyomas [10]. Additionally, low levels of Vit D and calcium can promote dysmenorrhea pain by elevating prostaglandin genesis or decreasing intestinal calcium absorption [11]. Moreover, a high Vit D intake may mitigate the risk of PMS, by effects on sex steroid hormone and neurotransmitter activities [12].

Curcumin (CUR) is a bioactive polyphenolic ingredient derived from the spice turmeric, acquired from the root of the *Curcuma longa* plant, a member of the Zingiberaceae family. CUR has a wide range of reported protective pharmacological properties such as anti-inflammatory, antioxidant, anti-neoplasm, neuro- and cardio-protective, immunomodulatory, analgesic, lipid-lowering and antidepressant effects [13–17]. CUR can interact with multiple molecular targets and modulate their activity including enzymes, inflammatory cytokines, transcription factors, growth factors, hormones, receptors, adipokines and various signaling cascades [16, 17]. One main advantage of CUR is related to safe and well-tolerable features in human. There are few studies indicated the potential therapeutic effect of CUR among PMS and dysmenorrhea women [18–21].

Correlation between dietary Vit D intake and the major circulating Vit D form, 25-hydroxyvitamin D [1,25-(OH)2D3], are relatively low [22] indicating that many factors modulate Vit D metabolism and absorption [23]. Results from a limited number of in vitro studies indicated that CUR potentially can directly or indirectly interact with vitamin D receptors (VDR) and its molecular targets [24–26].

Previously, we have shown the potential benefits on Vit D supplementation in the management of PMS and dysmenorrhea [2]. There are no clinical trials that have assessed the effects of CUR intervention on serum Vit D levels. Regarding to the high prevalence of menstrual problems, this randomized clinical trial performed in an attempt to evaluate the safety and effectiveness of CUR on Vit D in women suffered from both PMS and dysmenorrhea.

## Methods

### Study design

This study was a 3-month, triple-blind, randomized, placebo-controlled trial. A statistician provided a randomized list by using NCSS (statistical software) in a ratio of 1:1 using simple block randomization approach based on CONSORT guidelines. It was done concerning to standard guidelines, and was registered at the Iranian Registry of Clinical Trial (IRCT20191112045424N1 on 23/01/2021; available at: https://www.irct.ir). The protocol was approved by the by the Ethics Committee of BUMS (Code: IR.BUMS.REC.1398.160) and written informed consent was obtained by all participants. Patients were recruited from dormitories of 4 different universities in Birjand, South-Eastern of Iran, from January 2020 to March 2020.

Sample size was calculated according to α = 0.05, β = 0.2 and 95% confidence interval using the following formula and it was estimated that at least 25 patients were needed for each arm, and the final sample size assuming 15% drop-out rate was set as 30 patients in each group.

$$n=\frac{{\left({z}_{1-\frac{\alpha }{2}}+{z}_{1-\beta}\right)}^2\left({S}_1^2+{S}_2^2\right)}{{\left(\overline{x_1}-{\overline{x}}_2\right)}^2}$$

The eligible volunteers were single females aged 18 and 24 years and had a history of moderate to severe PMS and dysmenorrhea. Individuals were excluded if they experience any of the following situations: (a) any abnormal evidence on physical assessment, pelvic examination, as well as history of any acute or chronic illness, or drug and supplement use; (b) visual analog scale (VAS) score<8; (c) Premenstrual Syndrome Screening Tool (PSST) score <20; (d) getting married during trial; (e) irregular menstrual cycle; (f) allergy to herbal medicine; and (g) experiencing any stressful events during the intervention.

### Intervention

Participants were randomly allocated to the CUR group (*n* = 38) or placebo group (*n* = 38). Each CUR capsule contained 500 mg curcuminoids (C3 Complex, Sami Labs Ltd., Bangalore, India) plus piperine (5 mg Bioperine®, Sami Labs Ltd). Piperine as bioactive alkaloid was used to increase the oral bioavailability and intestinal absorption of CUR [27]. The placebo capsule was comparable in size, shape, color and texture did not contain curcuminoids, but containing 500 mg lactose powder in combination of piperine (5 mg Bioperine®, Sami Labs Ltd). CUR and placebo capsules were labeled as “code A” or “code B”. The Pharmacy Department of the Birjand University of Medical Science performed the randomization and blinding. Next, the eligible volunteers were randomly assigned to one of the two arms “code A or B”. Coding keys were forwarded to the principal study investigator through mail by the end of follow-up and final analysis. Participants were instructed to consume one capsule daily, for 10 days (7 days pre until 3 days after the estimated onset of menstruation) for 3 menstrual cycles. To promote adherence, we remember capsule consuming time to each subject by telephone and short message.

### Diagnosis of PMS and dysmenorrhea

A validated questionnaire was used to assess the degree of dysmenorrhea pain [28]. The VAS is a subjective evaluation of the pain on a point of 0 (no painful symptoms) to 10 (most severe pain possible), measurable in millimeters on a linear scale. Dysmenorrhea pain was classified on VAS as none (point: 0), mild (point: 1–3), moderate (point: 4–7), or worst imaginable pain (point: 8–10) [29].

Symptom severity of PMS was investigated with the use of the PSST questionnaire. The PSST is a 19 items related to different premenstrual symptoms which rated from 0 to 3, where score 0 refers “none”, and 3 shows the “severe”. Volunteer were instructed to choose a single number option from the scale for each item and total score obtained ranging from 0 to 57. A Persian language version of the PSST questionnaire has been previously indicated to be a reliable and valid method for the evaluating presence and intensity of PMS [30].

The subjects who obtained scores ≥8 (form VAS) and ≥ 20 (from PSST) were considered as having both PMS and dysmenorrhea and eligible to take part in the present study.

### Vitamin D measurement

Ten mLs of blood was collected into plain tubes, after overnight fasting 3 days prior to the start of intervention and within 3 days subsequent to the taking last capsule. Serum samples were separated and stored − 70 °C in a reference laboratory until laboratory analysis.

Serum Vit D (25-hydroxyvitamin D) was determined using an enzyme-linked immunosorbent assay (ELISA kit, Diazist, Tehran, Iran) based on the manufacturer’s protocol. Vit D status was categorized based on serum concentrations of 25(OH) D as follows: Vit D deficiency (< 20 ng/ml), and insufficiency (20–30 ng/ml) and sufficiency (> 30 ng/ml) [31].

### Anthropometric indices and blood pressure

Height, weight, waist and circumference, and systolic and diastolic pressure were measured by trained study nurses at in health center using standard protocol, as described previously [32]. Body mass index (BMI) was calculated as “weight (kg)/height ^2^(m).

### Dietary intake

The dietary intake of study participant was assessed by a trained dietician using a 3-day recall food method at the first week and last week of the study. Diet plan4 software was recruited for estimating of daily mean of energy and micronutrient intake throughout the trial (Forestfield Software Ltd., UK).

### Safety

To monitor safety and evaluation of adverse reactions, serum levels of urea, creatinine, alanine transaminase (ALT), aspartate transaminase (AST), alkaline phosphatase (ALP), total bilirubin, direct bilirubin, calcium, phosphate, magnesium and uric acid were measured. The serum levels of fasting blood glucose (FBG), urea, creatinine, calcium, phosphate, magnesium, uric acid, low density lipoprotein-cholesterol (LDL-C), high density lipoprotein-cholesterol (HDL-C), triglyceride (TG), total cholesterol (TC), ALT, AST, ALP, total bilirubin and direct bilirubin were measured by using commercial kits (Pars Azmun, Iran) with auto-analyzer (Prestige 24i, Tokyo Boeki Ltd., Tokyo, Japan) [33].

### Blinding

The participants, investigators, laboratory technicians, and statistical analyzer were completely blind to the treatment assignments during the trial until final analysis.

### Statistical analysis

The normality of continuous data were evaluated by the Kolmogorov-Smirnov test. Data were expressed as mean ± SD or median and interquartile range (IQR), as appropriate. The variables were compared between the two CUR and placebo groups by using student T test (parametric data) or Mann-Whitney (non-parametric data) or chi-square tests (for qualitative indices). Changes from the before to after-intervention within the groups determined by employing paired T test (normally distributed parameters) or Wilcoxon signed-rank test (non-normally distributed and categorical parameters). Improvement in Vit D status defined as percent of individuals get better from Vit D deficiency to insufficiency/sufficiency or from Vit D insufficiency to sufficiency. Changes in serum Vit D levels considered were the difference between the level of Vit D before and after supplementation. The statistical significance of the any independent effects of interventions on the main parameters was identified through an analysis of covariance (ANCOVA) taking the baseline value of each variable as a covariate. All statistical calculations were performed with SPSS software version 18 and *p* value < 0.05 was recruited to report data.

## Result

**Fig. 1:**
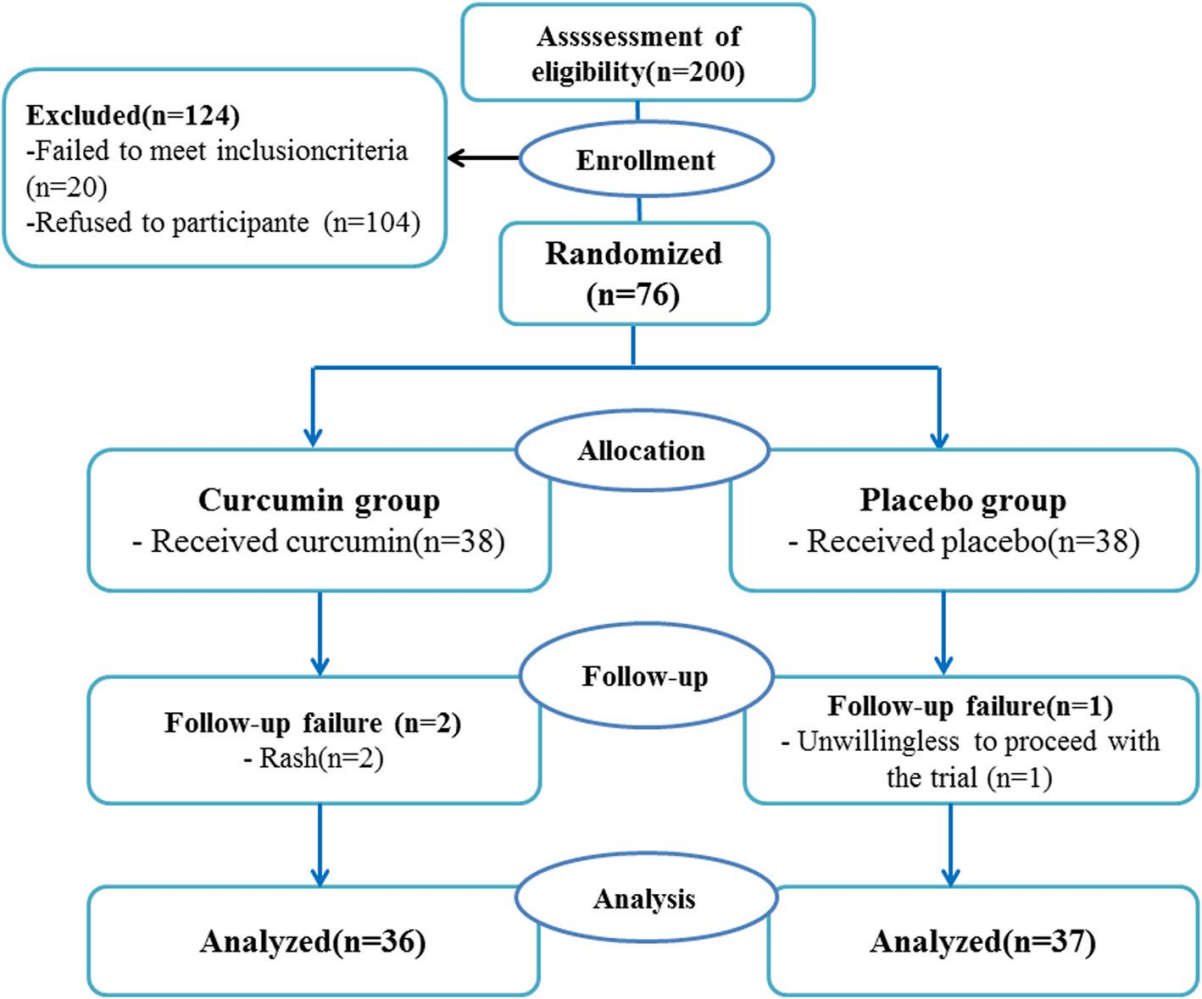
Flowchart of trial

Of the participants, 73 completed the study with CUR (*n* = 36) or placebo (*n* = 37). Three participants were withdrawn throughout follow-up due to rash side effects (*n* = 2; in CUR group) and unwillingness to continue the trial because of the personal reason (*n* = 1; in the placebo group). The drop-out rate was not statistically significant between the two groups (Fig. 1; *p* = 0.07).

The mean BMI, waist to hip ratio, systolic and diastolic blood pressure, TG, TC, HDL-C, LDL-C and FBG at baseline and end of the study were not statistically different between the two CUR and placebo groups (*P* > 0.05; Table 1). Statistical analysis could not detect any significant differences between two CUR and placebo groups concerning to the mean energy, or the dietary intake of carotene, vitamin E, vitamin C, calcium, phosphorus, magnesium, manganese, selenium, iron, zinc, vitamin A, and thiamin at baseline and end of the study (*P* > 0.05; Table 2). To monitor possible side effects of CUR consumption, we determined kidney function (urea and creatinine), liver function biomarkers (ALT, AST, ALP, total bilirubin, direct bilirubin), calcium, phosphate, magnesium and uric acid. The paired-sample T test showed no significant difference at the end-of-trial in terms of mean urea, creatinine, calcium, phosphate, magnesium, and uric acid in the studied groups (*P* > 0.05; Table 3). Serum levels of AST, and direct bilirubin were reduced by the end of trial in the CUR group (*P* = 0.040 and *P* = 0.021, respectively), but did not change in the control group (*p* > 0.05; Table 3).

**Table 1:** Comparison of biomarkers of traditional cardiovascular risk factors in treatment groups before and after intervention

Variables	Measurement period	Curcumin group (*n* = 36)	Placebo group (*n* = 37)	***P***^**a**^
BMI (kg/m2)	Before intervention	21.3 ± 2.3	20.8 ± 3.9	0.56
	After intervention	21.1 ± 2.1	21.0 ± 4.2	0.32
	*P*^*b*^	0.44	0.55	
WHR	Before intervention	0.73 ± 0.05	0.74 ± 0.05	0.51
	After intervention	0.75 ± 0.06	0.78 ± 0.12	0.31
	*P*^*b*^	0.19	0.07	
SBP (mmHg)	Before intervention	107.1 ± 9.6	105.8 ± 11.0	0.85
	After intervention	104.3 ± 9.2	103.8 ± 10.6	0.93
	*P*^*b*^	0.16	0.40	
DBP (mmHg)	Before intervention	73.5 ± 9.3	70.4 ± 7.0	0.65
	After intervention	71.0 ± 11.1	70.0 ± 10.0	0.68
	*P*^*b*^	0.20	0.83	
HDL-C (mg/dl)	Before intervention	49.8 ± 10.5	51.2 ± 8.4	0.15
	After intervention	49.9 ± 9.9	50.0 ± 9.2	0.46
	*P*^*b*^	0.92	0.25	
LDL-C (mg/dl)	Before intervention	73.6 ± 13.8	69.4 ± 17.1	0.43
	After intervention	78.2 ± 15.9	70.0 ± 16.3	0.10
	*P*^*b*^	0.11	0.72	
TG (mg/dl)	Before intervention	77.1 (55.2–90.7)	77.2 (49.5–86.5)	0.53
	After intervention	88.3 (61.2–99.5)	76.5 (56.2–83.2)	0.41
	*P*^*b*^	0.18	0.63	
TC (mg/dl)	Before intervention	154.7 ± 23.4	148.1 ± 25.1	0.47
	After intervention	159.0 ± 24.1	141.0 ± 33.6	0.051
	*P*^*b*^	0.27	0.16	
FBG (mg/dl)	Before intervention	84.4 ± 6.96	84.7 ± 8.15	0.92
	After intervention	86.5 ± 7.67	85.9 ± 7.55	0.74
	*P*^*b*^	0.24	0.31	

-Values expressed as mean ± SD (normally distributed variables) or median and interquartile range (non-normally distributed variables)

^a^*p* values indicate comparison between groups by using independent sample t test (normally distributed variables) or Mann-Whitney (non-normally distributed variables) at baseline and ANCOVA test after treatment

^b^*p* values indicate comparison within groups by using paired-sample T test (normally distributed variables) or Wilcoxon test (non-normally distributed variables)

*Abbreviations*: *BMI* Body mass index, *WHR* Waist to hip ratio, *SBP* Systolic blood pressure, *DBP* Diastolic blood pressure, *LDL-C* Low density lipoprotein-cholesterol, *HDL-C* High density lipoprotein-cholesterol, *TG* Triglyceride, *TC* Total cholesterol, *FBG* Fasting blood glucose

**Table 2:** Comparison of main micronutrient dietary intake of participants in treatment groups before and after intervention

Micronutrient	Measurement period	Curcumin group (*n* = 36)	Placebo group (*n* = 37)	***P***^**a**^
Energy (Kcal)	Before intervention	2169 ± 724	2103 ± 778	0.61
	After intervention	2120 ± 695	2102 ± 766	0.92
	*P*^*b*^	0.82	0.95	
Carotene (mcg)	Before intervention	691 ± 297	760 ± 392	0.33
	After intervention	709 ± 350	753 ± 404	0.49
	*P*^*b*^	0.76	0.82	
Vitamin E (mg)	Before intervention	13.1 ± 7.0	13.3 ± 7.3	0.85
	After intervention	13.0 ± 8.1	12.5 ± 6.9	0.54
	*P*^*b*^	0.89	0.62	
Vitamin C (mg)	Before intervention	68.1 ± 41.6	69.6 ± 35.2	0.92
	After intervention	63.5 ± 53.5	68.2 ± 30.0	0.74
	*P*^*b*^	0.79	0.92	
Calcium (mg)	Before intervention	207 ± 114	240 ± 141	0.13
	After intervention	221 ± 99	246 ± 162	0.47
	*P*^*b*^	0.68	0.83	
Phosphorus (mg)	Before intervention	407 ± 129	476 ± 139	0.19
	After intervention	442 ± 121	452 ± 161	0.92
	*P*^*b*^	0.69	0.75	
Magnesium (mg)	Before intervention	101 ± 38.1	98.9 ± 37.2	0.78
	After intervention	123 ± 22.9	95.4 ± 40.6	0.26
	*P*^*b*^	0.75	0.88	
Manganese (mg)	Before intervention	1.35 ± 0.54	1.59 ± 0.66	0.56
	After intervention	1.54 ± 0.98	1.46 ± 0.71	0.73
	*P*^*b*^	0.36	0.29	
Selenium (mcg)	Before intervention	22.9 ± 7.1	21.3 ± 9.4	0.41
	After intervention	23.2 ± 6.9	20.5 ± 7.8	0.47
	*P*^*b*^	0.87	0.69	
Iron (mg)	Before intervention	4.2 ± 1.3	4.0 ± 1.7	0.33
	After intervention	3.9 ± 1.7	3.8 ± 2.0	
	*P*^*b*^	0.66	0.61	
Zinc (mg)	Before intervention	2.4 ± 0.91	2.3 ± 1.8	0.23
	After intervention	2.6 ± 0.85	2.6 ± 1.2	0.93
	*P*^*b*^	0.80	0.87	
Vitamin A (RE)	Before intervention	42.6 ± 37.8	52.8 ± 56.1	0.22
	After intervention	39.8 ± 41.2	53.9 ± 49.3	0.15
	*P*^*b*^	0.76	0.93	
Thiamin (mg)	Before intervention	0.34 ± 0.20	0.39 ± 0.21	0.61
	After intervention	0.41 ± 0.23	0.37 ± 0.25	0.70
	*P*^*b*^	0.49	0.86	
Niacin (mg)	Before intervention	7.9 ± 2.4	6.1 ± 4.7	0.17
	After intervention	8.3 ± 3.1	6.8 ± 2.3	0.09
	*P*^*b*^	0.81	0.69	

-Values expressed as mean ± SD and adjusted for energy intake

^a^*p* values indicate comparison between groups by using independent sample t test

^b^*p* values indicate comparison within groups by using paired-sample T test

**Table 3:** Comparison of biochemical measures in treatment groups before and after intervention

Variables	Measurement period	Curcumin group (*n* = 36)	Placebo group (*n* = 36)	***P***^**a**^
Urea (mg/dl)	Before intervention	31.3 ± 7.9	29.8 ± 6.9	0.40
	After intervention	30.5 ± 6.9	28.4 ± 6.4	0.35
	*P*^*b*^	0.40	0.077	
Creatinine (mg/dl)	Before intervention	0.95 ± 0.08	1.03 ± 0.50	0.35
	After intervention	0.95 ± 0.07	1.12 ± 1.18	0.19
	*P*^*b*^	0.63	0.68	
ALT (IU/L)	Before intervention	17.9 ± 17.9	18.9 ± 6.1	0.49
	After intervention	17.7 ± 3.5	18.8 ± 6.1	0.37
	*P*^*b*^	0.66	0.91	
AST (IU/L)	Before intervention	16.1 ± 14.3	15.7 ± 5.8	0.87
	After intervention	10.2 ± 6.3	13.7 ± 12.2	0.14
	*P*^*b*^	**0.040**	0.29	
ALP (IU/L)	Before intervention	189.7 ± 37.7	196.2 ± 50.2	0.55
	After intervention	187.2 ± 37.2	187.7 ± 50.9	0.24
	*P*^*b*^	0.48	0.063	
Direct bilirubin (mg/dl)	Before intervention	0.27 ± 0.14	0.34 ± 0.16	0.053
	After intervention	0.22 ± 0.09	0.31 ± 0.13	**0.024**
	*P*^*b*^	**0.021**	0.13	
Total bilirubin (mg/dl)	Before intervention	0.54 ± 0.21	0.68 ± 0.34	0.051
	After intervention	0.48 ± 0.20	0.68 ± 0.47	0.23
	*P*^*b*^	0.11	0.96	
Calcium (mg/dl)	Before intervention	10.2 ± 0.42	10.3 ± 0.43	0.40
	After intervention	10.2 ± 0.35	10.2 ± 0.43	0.38
	*P*^*b*^	0.94	0.17	
Phosphate (mg/dl)	Before intervention	5.48 ± 0.63	5.15 ± 0.74	0.057
	After intervention	5.54 ± 0.70	5.27 ± 0.75	0.59
	*P*^*b*^	0.64	0.26	
Magnesium (mg/dl)	Before intervention	2.27 ± 0.32	2.23 ± 0.21	0.61
	After intervention	2.25 ± 0.25	2.20 ± 0.23	0.49
	*P*^*b*^	0.63	0.19	
Uric acid (mg/dl)	Before intervention	3.27 ± 0.92	3.35 ± 0.86	0.35
	After intervention	3.19 ± 0.73	3.13 ± 0.71	0.91
	*P*^*b*^	0.59	0.15	

-Values expressed as mean ± SD (normally distributed variables) or median and interquartile range (non-normally distributed variables)

^a^*p* values indicate comparison between groups by using independent sample t test (normally distributed variables) or Mann-Whitney (non-normally distributed variables) at baseline and ANCOVA test after treatment

^b^*p* values indicate comparison within groups by using paired-sample T test (normally distributed variables) or Wilcoxon test (non-normally distributed variables)

CUR significantly increased the median (IQR) serum levels of Vit D [from 12.8 ng/ml)7.0–24.6) to 16.2 ng/ml (6.4–28.8); *P* = 0.045], compared with placebo [from 18.6 ng/ml (2.2–26.8) to 21.3 ng/ml (5.2–27.1); *P* = 0.17; Table 4]. Whilst the percentage of individuals showing improvement in Vit D status at the end-of-trial, was significantly higher in the CUR group versus to baseline (*p* = 0.039), when compared with the placebo group, this was not statistically significant (25% vs. 18%; *p* = 0.71).

**Table 4:** Effect of curcumin vs. placebo on the vitamin D status and levels

Variables		Curcumin (*n* = 36)				Placebo (*n* = 37)				*P*
		Before	After	Improved	*P*	Before	After	Improved	*P*	
Vitamin D status	Deficiency	24 (66.7%)	18 (52.0%)	25%	**0.039**^**a**^	21 (56.8%)	17 (46.0%)	18%	0.08 ^**a**^	0.71^**b**^
	Insufficiency	7 (19.4%)	10 (27.8%)			8 (21.6%)	12 (32.4%)			
	Sufficiency	5 (13.9%)	8 (20.2%)			8 (21.6%)	8 (21.6%)			
Vitamin D levels (ng/ml)		12.8 (7.0 to 24.6)	16.2 (6.4 to 28.8)	2.2(−3.7 to 10.1)	**0.045**^**a**^	18.6 (2.2–26.8)	21.3 (5.2–27.1)	1.2(−2.0 to 6.0)	0.17 ^**a**^	**0.050**^**c**^

Data presented as number (percent) or Median (interquartile range)

^a^Comparison of before vs. after values in each group (Wilcoxon test)

^b^Comparison of before vs. after values between groups (chi-square test)

^c^Comparison of before vs. after values between groups (Man-Whitney test)

## Discussion

In this randomized triple-blind placebo controlled study we found that supplementation of curcuminoids plus piperine for three menstrual cycles, significantly improved the vitamin D status in women with PMS and dysmenorrhea.

Although the facts demonstrated that complementary and herbal medicines are overall preferable tolerated compared to chemical medications [34–36], in the absence of conclusive evidence about their efficacy and probable side effects on human disorders, made it one of the most critical obstacles faced through physician [37]. There is accumulating evidence demonstrating that CUR exhibits antioxidant, anti-inflammatory [38], antimicrobial [39], and anticarcinogenic [40] characteristics.

It has been suggested that imbalance of the renin-angiotensin-aldosterone system cascade is implicated in premenstrual fluid retention, with manifestations such as abdominal distension, limbs swelling, and breast discomfort [41, 42]. Vit D inadequacy is also connected with elevated RAAS activity, leading to higher fluid balance, blood pressure alterations, and hypertension [43]. Moreover, an inverse relationship was found between serum 25(OH) D amounts and premenstrual depression [44].

Results of the present trial supported that CUR intervention improves serum Vit D levels. But, Xin et al. reported that, although CUR administration had no considerable effect on serum 1,25-(OH)2D3 concentrations, it induced over-expression of VDR in femurs and osteoblasts, which might implicate in the protective effect of CUR from bone loss created by microgravity [24]. VDR as a nuclear transcription factor can modulate the activity of 1,25-(OH)2D3, so has important effect on calcium absorption, bone regeneration, and mineralization rate [45]. VDR is also expressed in the endometrium, ovarian tissue, fallopian tube epithelial cells and placenta. It also reported that CUR can directly binds and stimulates VDR, thus triggering the VDR target genes such as cytochrome P450 (CYP) 3A4, CYP24 and TRPV6 [26]. Interestingly, there is a remarkable overlap between 1,25-(OH)2D3 and CUR regarding to the molecular targets such as NF-κB and P21 [46–49]. However, findings of another experimental study did not support CUR act as VDR ligands [50]. Regarding the contribution of Vit D in the etiopathology of PMS and dysmenorrhea, its increment by CUR could represent a plausible mechanism for the beneficial therapeutic effect of CUR in menstrual-associated symptoms. Thus far, no human study has examined the consequence of CUR supplementing on serum Vit D status. This is also the first report on the effect of CUR on Vit D levels in patients with PMS and dysmenorrhea.

Another important result of the present study was effective decrement of serum AST and direct bilirubin following CUR intervention. Liver serum transaminases, ALT and AST, are generally considered as indicators of hepatic function and their elevation in blood associates to liver disease. Meta-analysis of four randomized controlled trials among 228 cases demonstrated a significant trend toward decrement of ALT and AST circulating concentrations in studies with ≥1000 mg daily CUR supplementation and in trials with 8-weeks intervention, respectively [51]. In the present study, CUR treatment was associated with a significant reduction in the serum levels of AST and direct bilirubin, suggesting that CUR may affect liver health even in healthy subjects [52]. Although, a significant reduction in ALT, ALP and total bilirubin levels after CUR supplementation was not detected in our study. Although this study has shown some promising results, individual health status and relatively low sample size in this research restricts the generalization of our findings to other population especially hepatic disorders.

There are different possible mechanisms which speculate beneficial effect of CUR on hepatic function. CUR might prevent hepatic steatosis, liver damage, insulin resistance [53], hepatic inflammation and fatty liver disease [54]. Furthermore, CUR suppresses high mobility group box 1 (HMGB1) and nuclear factor kappa B (NF-κB) induction, down-regulation of ICAM-1, cyclooxygenase-2 and MCP-1, decreases gene expression of pro-inflammatory cytokines, CD11b, procollagen type I, and tissue inhibitor of metalloprotease-1 and induction of peroxisome proliferator activated receptor-gamma (PPAR-γ) causing to the amelioration of the development and enhancement of inflammation in liver tissues and fibrosis [55, 56]. Also, antioxidant capacity of CUR is related with triggering several anti-oxidant enzymes activities such as glutathione transferase, catalase and heme-oxygenase-1 [57, 58].

The present results reveal that mean TG, TC, LDL-C and HDL-C concentrations did not show significant differences between the CUR and placebo groups. In agreement with our findings, Baum and co-workers announced CUR treatment did not significantly change serum TC or TG levels among elderly cases in a 6 months double blind, placebo-controlled trial [59], inconsistent to previous studies advocated the hypolipidemic effects of CUR in animals and humans [60, 61]. This controversy results between studies possibly because of various background diseases of the study participants (patients with acute coronary syndrome with LDL more than 150 mg/dl, individuals with cognitive impairment, patients with metabolic syndrome and healthy subjects) [59, 62–65].

Furthermore, CUR supplementation did not significantly change FBG levels in this triple-blind, placebo-controlled trial. Along with our results, several reports have been expressed null effect of CUR on glucose homeostasis, which contrary to previous studies supported the glucose-decreasing effects in humans [61, 66, 67]. The dosage of previous trials (ranging from 1500 to 6000 mg/day) was higher compared to the dosage administrated in this trial (500 mg/day), as well as treatment duration and formulation was also various. These discrepancies could be a probable interpretation of our finding. Furthermore, this study was not primarily aimed to assess the anti-diabetic and anti-lipid potency of CUR, and apparent healthy subjects constituted our studied population.

CUR had no significant effect on kidney function test in the present study. Consistently, serum levels of urea and creatinine not differed significantly comparing the pre- and post-turmeric supplementation in two clinical trials among patients with type 2 diabetic nephropathy [68] and chronic kidney disease [69].

The CUR has a very low water-solubility bioavailability, though, piperine, the active ingredient of black pepper, has been reported to boost the circulating levels and half-life of CUR in the body by 2000% through free-radical scavenging as well as preventing the lipid peroxidation and glucuronidation of xenobiotics [70].

This was a sub-study of our previous triple-blinded randomized controlled trial on short-term effects CUR supplementation on menstrual-associated symptoms and severity of PMS and dysmenorrhea in young women [71]. This study had some limitations. It is possible that 3 menstrual cycle intervention there is insufficient period to affect the biochemical markers evaluated in this trial. Short duration of follow-up was also inhibits judgment on the long-term effect of CUR supplementation particularly in terms of Vit D and liver function enzyme in young girl. Due to the rather small sample size and short-duration of the intervention, explanation of our findings should be made cautiously. Although, improving the Vit D levels does not necessarily reflect that CUR is effective against Vit D deficiency. So, further studies are needed to show whether CUR can have benefits on Vit D status.

## Conclusion

Taken together, our observations suggest that curcumin supplements in women with PMS and dysmenorrhea led to a significant improvement of Vit D, AST and direct bilirubin levels but did not affect blood glucose, uric acid, calcium, phosphorous and lipid profiles biomarkers. Future investigations are encouraged to look at possible dose-response association for the beneficial effect of curcuminoids on Vit D deficiency.

## Data Availability

The datasets used and analyzed during the current study are available from the corresponding author on reasonable request.

## References

[1] Bahrami A (2018). Menstrual disorders and premenstrual symptoms in adolescents: prevalence and relationship to serum calcium and vitamin D concentrations. J Obstet Gynaecol.

[2] Bahrami A (2018). High dose vitamin D supplementation can improve menstrual problems, dysmenorrhea, and premenstrual syndrome in adolescents. Gynecol Endocrinol.

[3] Shoaee F (2020). Evaluation of non-pharmacological strategies, therapeutic and cognitive-behavioral interventions in the treatment of premenstrual syndrome: a review study. Int J Pediatr.

[4] Bitzer J. Dysmenorrhea, premenstrual syndrome, and premenstrual dysphoric disorder, in Frontiers in Gynecological Endocrinology. Cham: Springer; 2014. p. 15–24.

[5] Ryan SA (2017). The treatment of dysmenorrhea. Pediatr Clin N Am.

[6] Rahnemaei FA, et al. Vitamin D supplementation for primary dysmenorrhea: a double-blind, randomized, placebo-controlled trial. Korean J Obstet Gynecol. 2021. 10.5468/ogs.20316 PMC8290151 34010550

[7] Binabaj MM (2018). The prognostic value of MGMT promoter methylation in glioblastoma: a meta-analysis of clinical trials. J Cell Physiol.

[8] Menon V (2020). Vitamin D and depression: a critical appraisal of the evidence and future directions. Indian J Psychol Med.

[9] Makrani AH (2017). Vitamin D and fibromyalgia: a meta-analysis. Korean J Pain.

[10] Corachán A (2021). Vitamin D as an effective treatment in human uterine leiomyomas independent of mediator complex subunit 12 mutation. Fertil Steril.

[11] Abdi F (2021). Role of vitamin D and calcium in the relief of primary dysmenorrhea: a systematic review. Obstet Gynecol Sci.

[12] Bertone-Johnson ER (2009). Vitamin D and the occurrence of depression: causal association or circumstantial evidence?. Nutr Rev.

[13] Bahrami A, Majeed M, Sahebkar A (2019). Curcumin: a potent agent to reverse epithelial-to-mesenchymal transition. Cell Oncol.

[14] Bahrami A, Ferns GA. Effect of curcumin and Its derivates on gastric cancer: molecular mechanisms. Nutr Cancer. 2020;73(9):1553–69. 10.1080/01635581.2020.1808232 32814463

[15] Bahrami A (2018). Genetic and epigenetic factors influencing vitamin D status. J Cell Physiol.

[16] Abdeahad H (2020). Clinical association between Phospho-AKT expression with clinicopathological characteristics of gastrointestinal cancer patients: a meta-analysis. Crit Rev Eukaryot Gene Expr.

[17] Parsamanesh N (2018). Therapeutic potential of curcumin in diabetic complications. Pharmacol Res.

[18] Khayat S (2015). Curcumin attenuates severity of premenstrual syndrome symptoms: a randomized, double-blind, placebo-controlled trial. Complement Ther Med.

[19] Fanaei H, Behboodi Moghadam Z, Kasaeiyan A (2015). Comparison the effects of ginger and curcumin in treatment of premenstrual syndrome. ISMJ.

[20] Kheirkhah M (2016). The effect of curcumin on premenstrual syndrome symptoms: a double-blind randomized clinical trial. Nurs Midwifery J.

[21] Rahman SF (2020). Influence of curcumin and ginger in primary dysmenorrhea: a review. Int J Appl Eng Res.

[22] Bertone-Johnson ER (2010). Dietary vitamin D intake, 25-hydroxyvitamin D3 levels and premenstrual syndrome in a college-aged population. J Steroid Biochem Mol Biol.

[23] Khajehei M (2009). Effect of treatment with dydrogesterone or calcium plus vitamin D on the severity of premenstrual syndrome. Int J Gynecol Obstet.

[24] Xin M (2015). Attenuation of hind-limb suspension-induced bone loss by curcumin is associated with reduced oxidative stress and increased vitamin D receptor expression. Osteoporos Int.

[25] Kim JH (2012). Turmeric (Curcuma longa) inhibits inflammatory nuclear factor (NF)-κB and NF-κB-regulated gene products and induces death receptors leading to suppressed proliferation, induced chemosensitization, and suppressed osteoclastogenesis. Mol Nutr Food Res.

[26] Bartik L (2010). Curcumin: a novel nutritionally derived ligand of the vitamin D receptor with implications for colon cancer chemoprevention. J Nutr Biochem.

[27] Shoba G (1998). Influence of piperine on the pharmacokinetics of curcumin in animals and human volunteers. Planta Med.

[28] Crichton N (2001). Visual analogue scale (VAS). J Clin Nurs.

[29] Osayande AS, Mehulic S (2014). Diagnosis and initial management of dysmenorrhea. Am Fam Physician.

[30] Siahbazi S (2011). Translation and psychometric properties of the Iranian version of the premenstrual symptoms screening tool (PSST). Payesh (Health Monitor).

[31] Holick MF (2007). Vitamin D deficiency. N Engl J Med.

[32] Ho S (2001). Association between simple anthropometric indices and cardiovascular risk factors. Int J Obes.

[33] Zargari M, Sedighi O. Influence of hemodialysis on lipid peroxidation, enzymatic and non-enzymatic antioxidant capacity in chronic renal failure patients. Nephrourol Mon. 2015;7(4):e28526. 10.5812/numonthly.28526 PMC4628136 26539417

[34] Bagherniya M (2018). Medicinal plants and bioactive natural compounds in the treatment of non-alcoholic fatty liver disease: a clinical review. Pharmacol Res.

[35] Colalto C (2018). What phytotherapy needs: evidence-based guidelines for better clinical practice. Phytother Res.

[36] Hadi A (2019). Effect of purslane on blood lipids and glucose: a systematic review and meta-analysis of randomized controlled trials. Phytother Res.

[37] Izzo AA (2016). A critical approach to evaluating clinical efficacy, adverse events and drug interactions of herbal remedies. Phytother Res.

[38] Boroumand N, Samarghandian S, Hashemy SI (2018). Immunomodulatory, anti-inflammatory, and antioxidant effects of curcumin. J Herbmed Pharmacol.

[39] Adamczak A, Ożarowski M, Karpiński TM (2020). Curcumin, a natural antimicrobial agent with strain-specific activity. Pharmaceuticals.

[40] Bahrami A, Ferns GA (2021). Effect of curcumin and its derivates on gastric cancer: molecular mechanisms. Nutr Cancer.

[41] Tollan A (1993). Evidence for altered transcapillary fluid balance in women with the premenstrual syndrome. Acta Obstet Gynecol Scand.

[42] Forman JP, Williams JS, Fisher ND (2010). Plasma 25-hydroxyvitamin D and regulation of the renin-angiotensin system in humans. Hypertension.

[43] Vaidya A, Forman JP (2012). Vitamin D and vascular disease: the current and future status of vitamin D therapy in hypertension and kidney disease. Curr Hypertens Rep.

[44] Eyles DW, Burne TH, McGrath JJ (2013). Vitamin D, effects on brain development, adult brain function and the links between low levels of vitamin D and neuropsychiatric disease. Front Neuroendocrinol.

[45] Pike JW, Yamamoto H, Shevde NK (2002). Vitamin D receptor–mediated gene regulation mechanisms and current concepts of vitamin D analog selectivity. Adv Ren Replace Ther.

[46] Szeto FL (2007). Involvement of the vitamin D receptor in the regulation of NF-κB activity in fibroblasts. J Steroid Biochem Mol Biol.

[47] Inoue Ji (2007). NF-κB activation in development and progression of cancer. Cancer Sci.

[48] Su C-C (2006). Curcumin-induced apoptosis of human colon cancer Colo 205 cells through the production of ROS, Ca2+ and the activation of caspase-3. Anticancer Res.

[49] Hour TC (2002). Curcumin enhances cytotoxicity of chemotherapeutic agents in prostate cancer cells by inducing p21WAF1/CIP1 and C/EBPβ expressions and suppressing NF-κB activation. Prostate.

[50] Guo C (2013). Curcumin induces human cathelicidin antimicrobial peptide gene expression through a vitamin D receptor-independent pathway. J Nutr Biochem.

[51] Mansour-Ghanaei F (2019). Efficacy of curcumin/turmeric on liver enzymes in patients with non-alcoholic fatty liver disease: a systematic review of randomized controlled trials. Integr Med Res.

[52] DiSilvestro RA (2012). Diverse effects of a low dose supplement of lipidated curcumin in healthy middle aged people. Nutr J.

[53] Tabrizi R (2018). The effects of curcumin on glycemic control and lipid profiles among patients with metabolic syndrome and related disorders: a systematic review and meta-analysis of randomized controlled trials. Curr Pharm Des.

[54] Egashira K (2012). Food-drug interaction of tacrolimus with pomelo, ginger, and turmeric juice in rats. Drug Metab Pharmacokinet.

[55] Leclercq IA (2004). Curcumin inhibits NF-κB activation and reduces the severity of experimental steatohepatitis in mice. J Hepatol.

[56] Wang Y (2014). Comparison between the efficacies of curcumin and puerarin in C57BL/6 mice with steatohepatitis induced by a methionine-and choline-deficient diet. Exp Ther Med.

[57] Iqbal M (2003). Dietary supplementation of curcumin enhances antioxidant and phase II metabolizing enzymes in ddY male mice: possible role in protection against chemical carcinogenesis and toxicity. Pharmacol Toxicol.

[58] Motterlini R (2000). Curcumin, an antioxidant and anti-inflammatory agent, induces heme oxygenase-1 and protects endothelial cells against oxidative stress. Free Radic Biol Med.

[59] Baum L (2007). Curcumin effects on blood lipid profile in a 6-month human study. Pharmacol Res.

[60] Arafa HM (2005). Curcumin attenuates diet-induced hypercholesterolemia in rats. Med Sci Monit.

[61] Panahi Y (2016). Curcumin lowers serum lipids and uric acid in subjects with nonalcoholic fatty liver disease: a randomized controlled trial. J Cardiovasc Pharmacol.

[62] Yang YS (2014). Lipid-lowering effects of curcumin in patients with metabolic syndrome: a randomized, double-blind, placebo-controlled trial. Phytother Res.

[63] Sahebkar A (2014). A systematic review and meta-analysis of randomized controlled trials investigating the effects of curcumin on blood lipid levels. Clin Nutr.

[64] Pungcharoenkul K, Thongnopnua P (2011). Effect of different curcuminoid supplement dosages on total in vivo antioxidant capacity and cholesterol levels of healthy human subjects. Phytother Res.

[65] Alwi I (2008). The effect of curcumin on lipid level in patients with acute coronary syndrome. Acta Med Indones.

[66] Chuengsamarn S (2012). Curcumin extract for prevention of type 2 diabetes. Diabetes Care.

[67] Na LX (2013). Curcuminoids exert glucose-lowering effect in type 2 diabetes by decreasing serum free fatty acids: a double-blind, placebo-controlled trial. Mol Nutr Food Res.

[68] Khajehdehi P (2011). Oral supplementation of turmeric attenuates proteinuria, transforming growth factor-β and interleukin-8 levels in patients with overt type 2 diabetic nephropathy: a randomized, double-blind and placebo-controlled study. Scand J Urol Nephrol.

[69] Moreillon JJ (2013). The use of an anti-inflammatory supplement in patients with chronic kidney disease. J Complement Integr Med.

[70] Srinivasan K (2014). Antioxidant potential of spices and their active constituents. Crit Rev Food Sci Nutr.

[71] Bahrami A, et al. Effects of curcumin on menstrual pattern, premenstrual syndrome, and dysmenorrhea: a triple-blind, placebo-controlled clinical trial. Phytother Res. 2021;35(12):6954–62. 10.1002/ptr.7314 34708460

